# Supplementary material for: Quantum dot LEDs emitting broadband vortex beams
Source: Nat Commun. 2025 May 29;16:4974. doi: 10.1038/s41467-025-60336-w (PMC12122666; doi:10.1038/s41467-025-60336-w)
Supplement: Supplementary file 1 — Supplementary Information [file 41467_2025_60336_MOESM1_ESM.pdf]

Quantum Dot LEDs Emitting Broadband Vortex Beams

Supplementary Information

Guillaume Boulliard[1], Iännis Roland[1], Domitille Schanne[1], Marie Petolat[1], Pascal Filloux[1], Emmanuel Lhuillier[2], Aloyse Degiron[1,*]

[1]Université Paris Cité, CNRS, Laboratoire Matériaux et Phénomènes Quantiques, 75013 Paris, France

[2]Sorbonne Université, CNRS, Institut des NanoSciences de Paris, 75005 Paris, France

[*]aloyse.degiron@u-paris.fr

1. Full description of the numerical simulations S2

2. Colloidal quantum dot synthesis S3

2.1 n-doped PbS QDs for light emission S3

2.2 p-doped PbS QDs for hole injection S4

3. LED fabrication S4

3.1 QD LEDs operating with Au patterns S4

3.2 QD LEDs operating with a-Si patterns S10

4. Supplementary data S12

5. Supplementary references S16

1. Full description of the numerical simulations

Away from the center of the samples, the spiral holograms can be approximated as linear gratings along any azimuthal direction. Therefore, we perform our full-wave simulations in two-dimensions, by simulating a periodic unit cell flanked by periodic boundary conditions using commercial code (COMSOL Multiphysics, RF module).

For QD LEDs operating with Au grating, the period is 1200 nm and the different domains are, from bottom to top:

- an Al cathode, with wavelength-dependent material parameters taken from Lynch and Hunter[1]. Since the fields cannot deeply penetrate in Al, the thickness of this layer is rather arbitrary. In our simulations, we take a thickness of 200 nm to ensure that no field ever reaches the bottom boundary of the domain (in reality, the thickness is 80 nm, see section 3 below).

- a 70 nm layer of mesoporous TiO_2, with a permittivity taken from ellipsometry measurements published elsewhere[2]. Importantly we found it necessary to replace the experimental imaginary part of this permittivity by a high value of 0.3i to reproduce the linewidths of the different branches apparent in the experimental dispersion relations. It is likely that these high material losses are actually provoked by the roughness of the mesoporous TiO_2 layer, made of self-assembled nanocrystals (see section 3 below). In our model, we model all layers with flat interfaces without roughness so we must account for the roughness-induced losses by artificially increasing the imaginary part of the permittivity.

- a single element of the Au grating, with a thickness of 25 nm (corresponding to the nominal thickness of Au deposited on the sample) and material parameters taken from[3]. In our structures, the duty cycle is 0.25 and the period is 1200 nm. Thus, the length of the Au element is 300 nm.

- A 250 nm thick PMMA layer (corresponding to the nominal thickness of PMMA deposited on the sample), with a refractive index of 1.47.

- Following a strategy that we successfully employed in past studies on functional LEDs[2,4], we merge the top three layers (respectively made of n-doped PbS QDs, p-doped PbS QDs and ITO) into a single effective layer with a thickness of 95 nm and a complex permittivity equal to 4.45+0.13i. The reason for this approximation is that we have a high experimental uncertainty on the actual thickness and refractive index of the few monolayers of PbS QDs deposited on the sample.

- The top layer is an air box with an electromagnetic port as upper boundary condition. This port is used to launch a plane wave and compute the reflectivity spectrum.

To compute the dispersion relation of this two-dimensional structure, we calculate the reflectivity spectrum for incidence angles varying from 0 to asin(0.65) ≈ 40.5°, corresponding to the collection angle of our experimental LCPLN50XIR microscope objective from Olympus with a numerical aperture of 0.65. The calculation is repeated twice—one for the TE polarization and one for the TM polarization. To compare the data with the unpolarized experimental dispersion (Fig. 1d), we sum the TE- and TM-polarized contributions. Then, we weight the result by the EL intensity of the experimental dispersion relation of the actual LED integrated over all $k_{//}/k_0$ values.

The numerical simulations for QD LEDs operating with a-Si holograms are performed with the same model, except that the Au grating is replaced by a 30-nm thick element with a refractive index n=3. Moreover, just as the actual LEDs, the periodicity is reduced to 1000 nm.

2. Colloidal quantum dot synthesis

Chemicals: Octadecene (ODE, Acros Organics, 90%), Lead oxide (PbO, Strem Chemicals, 99.999+%-), Oleic acid (OA, Alfa Aesar 90%), Hexamethyldisilathiane (TMS_2S, Sigma Aldrich, synthesis grade), lead chloride ($PbCl_2$, Alfa Aesar, 99%), sulfur powder (S, Alfa Aesar, 99.5%), Ethanol (VWR, >99.9%), n-Octane (SDS, 99%), n-Hexane (VWR, 99%), Toluene (Carlo Erba, >99.8%), 1,2 Ethanedithiol (EDT, Fluka, 98%), %), 3-Mercaptopropionic acid (MPA Aldrich).

2.1 n-doped PbS QDs for light emission

In a 50 mL three neck flask, 514 mg of $PbCl_2$ (1.85 mmol) and 7.5 mL of oleylamine were degassed under vacuum at 110°C for 60 min. Meanwhile, a solution of S powder (40 mg, 1.25 mmol) in 7.5 mL of OLA was sonicated until full dissolution: an orange clear solution was obtained. After the atmosphere was switched to N_2 and the temperature set at 90°C, the S solution was quickly injected. After 1 min, the reaction was quenched by injecting a mixture of 1 mL of OA and 10 mL of hexane. The content of the flask was transferred in a centrifuge tube and precipitated with ethanol to complete the volume up to 45 mL. After centrifugation, the formed pellet was redispersed with 3 mL of hexane and 1 drop of oleic acid. The cleaning

procedure was repeated one more time (V_{EtOH} = 2 mL, $V_{toluene}$ = 3 mL). At this step the nanocrystals were centrifuged in fresh toluene to get rids of undispersed nanocrystals. Filtration (0.2 µm).

2.2 p-doped PbS QDs for hole injection

The procedure is inspired from Hines *et al*[5]. 0.9 g of PbO are introduced in a 100 mL three neck flask with 3 g of OA and 47 g of ODE. The flask is degassed under vacuum at 120 °C for 2 hours. Meanwhile, in an air free glove box, a mixture of 420 µL of TMS_2S and 10 mL of ODE is prepared in a 20 mL vial, then introduced into a 20 mL syringe. The atmosphere of the flask is switched to Ar and the temperature is set equal to 90 °C. The TMS_2S solution is quickly injected and the solution turns dark while the temperature drops to 80 °C. After 8 min at 80 °C, the reaction is stopped by removing the heating mantle and prompt cooling of the flask by addition of a mixture of heptane and OA. The nanoparticles are then precipitated by addition of ethanol. The formed pellet is redispersed in toluene. A second step of cleaning is repeated. Finally, the pellet is redispersed in toluene with a 50 $mg.mL^{-1}$ concentration. The solution is centrifuged to remove any colloidally unstable material. Finally, the solution is filtered on a 0.22 µm PTFE filter.

3. LED fabrication

3.1 QD LEDs operating with Au patterns

- **Wafer preparation**

The first step of the fabrication is to coat a 2 inch Si wafer with 500 nm of SiO_2 deposited by plasma enhanced chemical vapor deposition (PECVD) using a Corial D250L PECVD system. The goal of this layer is to provide an insulating substrate for the LEDs that will be fabricated onto it.

- **Al cathodes deposition with photolithography and e-beam evaporation**

The Al cathodes, which are 500 micron wide, and defined by photolithography. In more details, we first spin-coat HMDS, an adhesion promoter, at 500 rpm for 3s followed by 5000 rpm for 30 s. After waiting for around one minute, we spin-coat S1818 resist at 500 rpm for 3s followed by 5000 rpm for 30 s. Then, we bake the wafer at 110°C for 60 s. An optical mask made of

chromium on quartz is used to transfer the cathode pattern on the resist with a 15s UV light exposition, using an MJB4 Süss MicroTec aligner equipped with a 200 W mercury UV lamp.

The exposed resist is developed by dipping the wafer in MF319 for 1 min, then in deionized water for 1 min before drying with nitrogen. The sample is then coated with 2 nm of Ti at 0.02 nm/s and 80 nm of Al at 0.1 nm/s using our MEB550S E-beam evaporator by PLASSYS. The remaining resist is then lifted in a bath of acetone for 30 min accompanied with ultrasounds for the last few seconds. Finally, the wafer is rinsed in isopropanol (IPA) for 1 min and dried with nitrogen.

- **Cleaving and cleaning the samples**

We cleave the 2 inch wafer into 1 cm x 1 cm squares that are subsequently cleaned with acetone, IPA and O_2 plasma for 5 minutes. The rest of the fabrication will be performed on one of these 1 cm x 1 cm squares. Four Al cathodes are present on each square, with a center-to-center separation distance of 2 mm.

- **Protecting the Al cathodes**

Before completing the LED stack, we protect one side of the Al cathodes with specialized tape. This step is necessary to prevent the cathodes from being entirely buried under the subsequent layers, which would make it impossible to contact them electrically.

- **TiO_2 deposition**

The sample is coated with a 60 nm thick layer of anatase TiO_2 nanocrystals (1.1 part of Ti-Nanoxide HT-L/SC from Solaronix diluted in 1 part of ethanol). To succeed, it is first necessary to expose the sample to UV ozone for 10 min to improve adhesion and to load it in a spin-coater filled with an ethanol-saturated atmosphere in order to minimize unwanted striations. We obtain this condition by covering the inside of the spin-coater with ethanol-soaked paper. 18 µL of the diluted TiO_2 nanocrystal solution is spun onto the sample at 5000 rpm for 60 seconds. The layer is then partially sintered by baking the sample at 200°C for 15 min.

- **Fabrication of the Au holograms using electron beam (e-beam) lithography**

We spin-coat 18 µL of CSAR62 resist (from Allresist) at 500 rpm for 3 s followed by 3300 rpm for 60 s on top of the TiO_2 layer, then bake it at 150°C for 60 s. The final thickness is around 80 nm.

TiO_2 is conductive enough to avoid charge accumulation during the writing process, thus avoiding the need to add a charge dissipative layer on top of the CSAR62 resist. The patterns are transferred in the resist with a Raith Pioneer II e-beam lithography system, with an acceleration of 20 kV and a nominal dose of 50 µC/cm². The writing time for each pattern is approximately 15 min. 3 structures are distributed along the length of each Al cathode (and given that there are four Al cathodes per sample, we fabricate a total of 12 independent LEDs on each substrate). In addition to the Au spiral gratings and Au interferometric patterns, we add labels to identify the different structures as well as alignment marks that will be used later to position the apertures in the PMMA layer at the center of each device. The exposed parts of the resist are developed in a bath of AR600-546 (Allresist) for 60 s, then in a mixture of ¼ of Methyl isobutyl ketone (MIBK) and ¾ IPA for 30 s, and finally in IPA for 30 s. Before blow-drying the sample with N_2, we found it essential to dip the sample in deionized water for 10 s to get consistent and reproducible electrical pumping. We then deposit 2 nm of Ti at 0.02 nm/s and 25 nm of Au at 0.02 nm/s, using the e-beam evaporator. The Au patterns are finally obtained after a lift-off in 2-butanone in an ultrasound bath at 50°C for 10 min, followed by a rinse in IPA for 30 s and blow-dry with N_2.

- **Insulating PMMA layer with apertures made by e-beam lithography**

A thickness of 250 nm of PMMA (950 PMMA A6 from Kayaku, further diluted in anisole) is applied on the sample by spin-coating. Then, a new round of e-beam lithography with an acceleration of 20 kV and a nominal dose of 220 µC/cm² is performed to define apertures with a radius of 1.5 µm at the center of each Au pattern. The exposed parts are subsequently eliminated by developing the sample in MIBK for 30 s followed by a 30 s rinse in IPA and a 30 s dip in H_2O. The rinse in H_2O removes the inorganic residues left by the organic solvents. We have discovered that it was a critical step for improving the reliability of the electrical injection, probably because the inorganic residues lead to unwanted band-bending and Fermi pinning effects. We do not lift the remaining PMMA, which simultaneously serves as an electrically insulating layer and as an essential waveguiding element within the LED stack. The PMMA thickness is a result of an empirical tradeoff: thinner layers were more sensitive to electrical leaks/shorts while thicker layers were detrimental to the next fabrication steps, the lithographied hole in the PMMA becoming too deep to ensure proper physical and electrical continuity between the ITO inside this hole and the rest of the ITO electrode.

- **Emissive layer made of n-doped PbS QDs cross-linked with 3-mercaptopropionic acid (MPA)**

The emissive layer is obtained by spin-coating 18 µl of n-doped PbS QDs (synthesis detailed in section 2.1) onto the sample at 3000 rpm for 60 s. The sample is then dipped in a solution of 1% of MPA (3-Mercaptopropionic acid, Aldrich**)** with ethanol, and then rinsed in pure ethanol, so as to remove the native OA ligands capping the QDs and to cross-link them with MPA instead. This procedure is then repeated a second time to increase the thickness of this layer. The size of the emissive quantum dots is ~4.5 nm and after ligand-exchange, the interdot spacing is on the order of 1 nm, corresponding to a density of ~ 6×10^6 QD/µm^3.

- **Hole transfer layer made of p-doped PbS QDs cross-linked with ethane-1,2-dithiol**

The fabrication of the hole transfer layer is performed in a N_2-filled glove box by spin-coating 18 µL of p-doped PbS QDs (synthesis detailed in section 2.2) at 4500 rpm for 60 s. We then perform a ligand exchange step without removing the sample from the spin-coater, by dropping 60 µl of a solution of 0.1% of EDT in ethanol on top of it. After waiting for 1 min, the sample is spun at 4500 rpm for 60s. We finally rinse the sample two times, each time by dropping 40 µl of ethanol on top of it and by spinning it at 4500 rpm for 60s.

- **Indium tin oxide (ITO) Anodes**

The last step of our fabrication process is the deposition of 80 nm of ITO by RF sputtering of an ITO target with an Ar plasma in an Emitech K675XD system. The deposition is made through a shadow mask, so as to create 500 µm wide ITO stripes that are oriented perpendicular to the Al cathodes. The shadow mask is positioned so that the ITO stripes are aligned with the different Au patterns of the stack. Following a well-established strategy[2,4,6], we actually deposit two layers of ITO. First, we deposit a layer made of small grains with a first cycle of sputtering at 100 mA for 50 s, so as to minimize the internal stress within the LED stack. Then, we deposit a higher conductivity layer made of larger grains with two consecutive sputtering cycles at 150 mA for 50 s.

The main steps of this fabrication process are summarized in Supplementary Fig. 1.

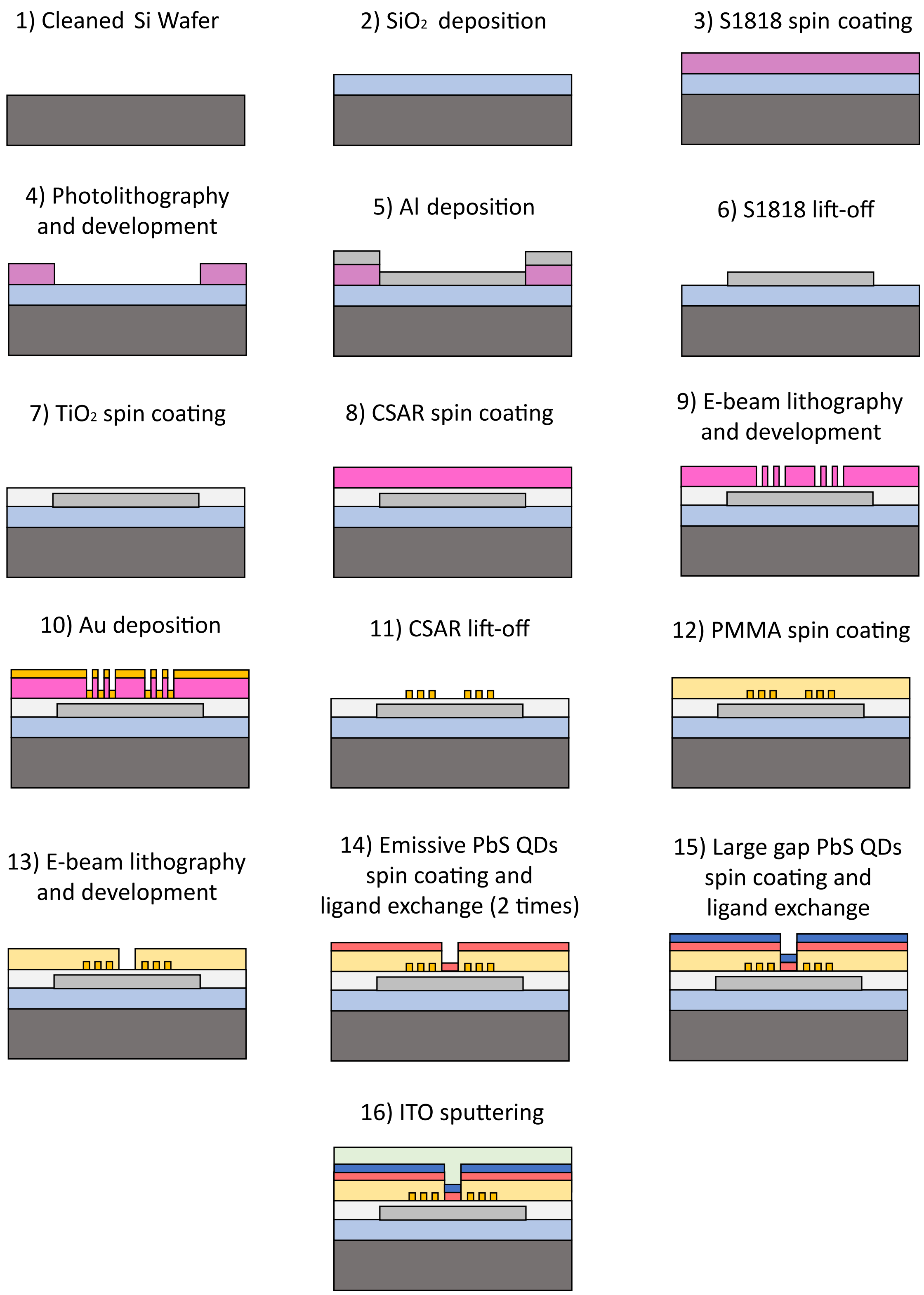

Supplementary Fig. 1 | Fabrication workflow of the QD LEDs operating with a Au hologram. The different steps are numbered from 1 to 16.

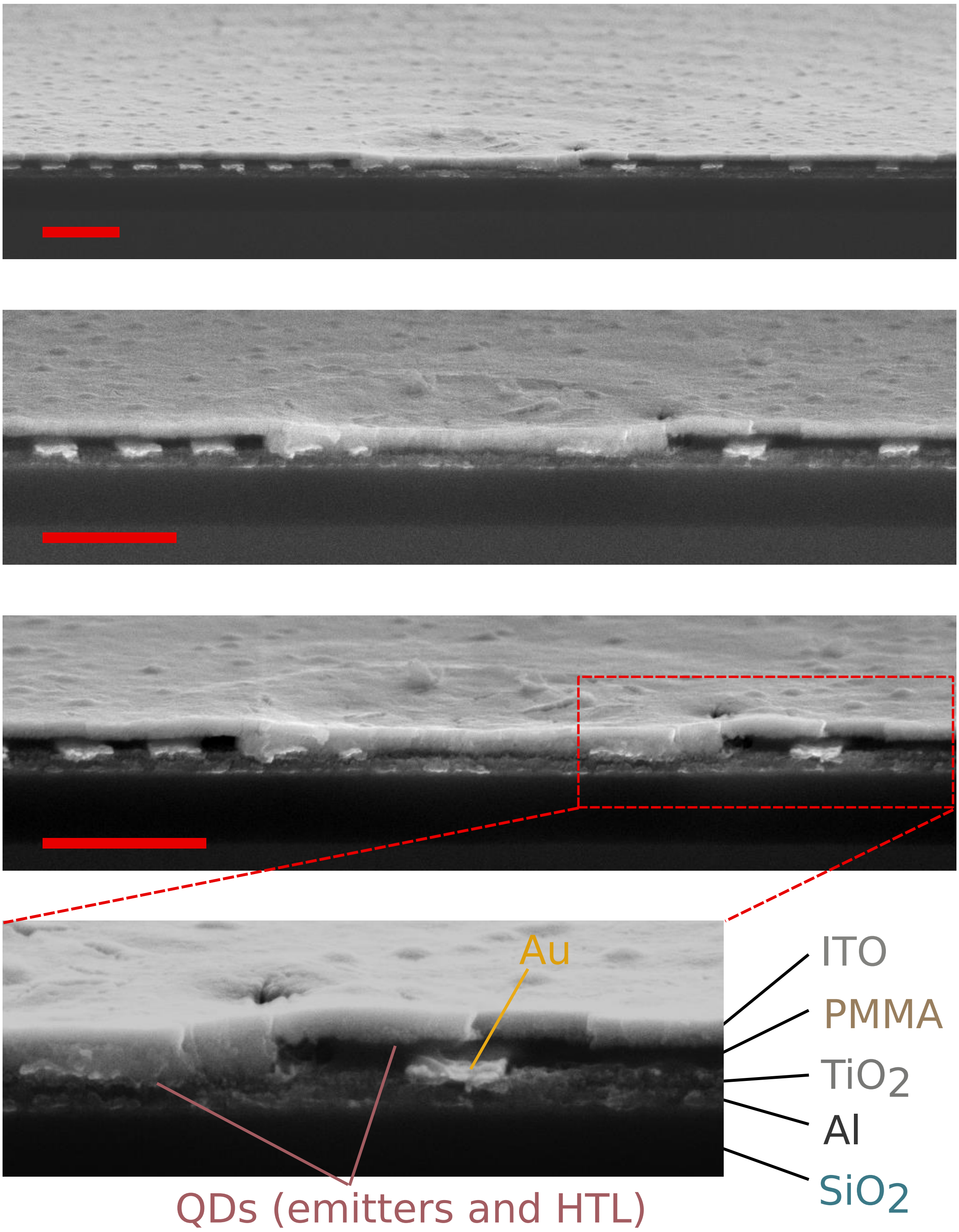

Supplementary Fig. 2 | Scanning electron micrographs showing a cross-section of one of our quantum dot LEDs at different magnifications. This LED operates with an interferometric Au pattern identical to the one producing the I_2 pattern of Fig. 2c, explaining why the periodicity of the Au stripes is not the same on each side of the central injection area without PMMA. The red scale bar represents 1 micron in the top three views. These views, taken at an angle of 80°, do not necessarily provide accurate information on the actual thickness of the different layers because each of them is cleaved in a slightly different plane, meaning that some layers are partially hiding others (this is particularly evident with the Au stripe of the bottom view, which seems to protrude from the cross-section, or with the ITO layer showing multiple cleaving planes).

3.2 QD LEDs operating with a-Si patterns

The fabrication process is essentially the same as for the QD LEDs operating with Au holograms, except for the definition of the a-Si patterns. The relevant steps are detailed here:

- **Deposition of 30 nm of a-Si**

We rely for this step on plasma-enhanced chemical vapour deposition (PECVD) in a Corial D250 system, with SiH_4 at a flow rate of 10 sccm and Ar at a flow rate of 1000 sccm. The pressure is set at 1400 mT, the RF power is 100 W and the temperature 280°C. The deposition time is 30 seconds.

- **Fabrication of a-Si holograms with e-beam lithography and reactive ion etching (RIE)**

The resist used for the e-beam lithography step is again CSAR 62 from Allresist, 18 µL of which are spun on top of the a-Si layer at 500 rpm for 3 s followed by 3300 rpm for 60 s. This time, we use this resist as a mask that will protect the unexposed parts of the resist from the subsequent etching step. For this reason, the e-beam lithography patterns are inverted compared to those used in Au. These patterns are written with an acceleration of 20 kV and a nominal dose of 50 µC/cm^2, along with labels and alignment marks that will be used in later steps.

After e-beam writing, we develop the exposed parts of the resist in a bath of AR600-546 for 60 s, then in a mix of ¼ of MIBK and ¾ IPA for 30s, then in IPA for 30 s and finally deionized water for 10 s. We then remove the unprotected parts of the a-Si layer in a Corial 200R system for 1 min with CHF_3 at a flow rate of 50 sccm, O_2 at a flow rate of 3 sccm, the power set at 150 W, the temperature at 20°C, and the pressure at 50 mTorr.

We then remove the CSAR 62 mask by soaking the sample in 2-butanone subject to an ultrasonic bath at 50°C for 10 min. After a rinsing dip in IPA for 30 seconds, the sample is finally dried with N_2.

The main steps of the whole fabrication process are summarized in Supplementary Fig. 3.

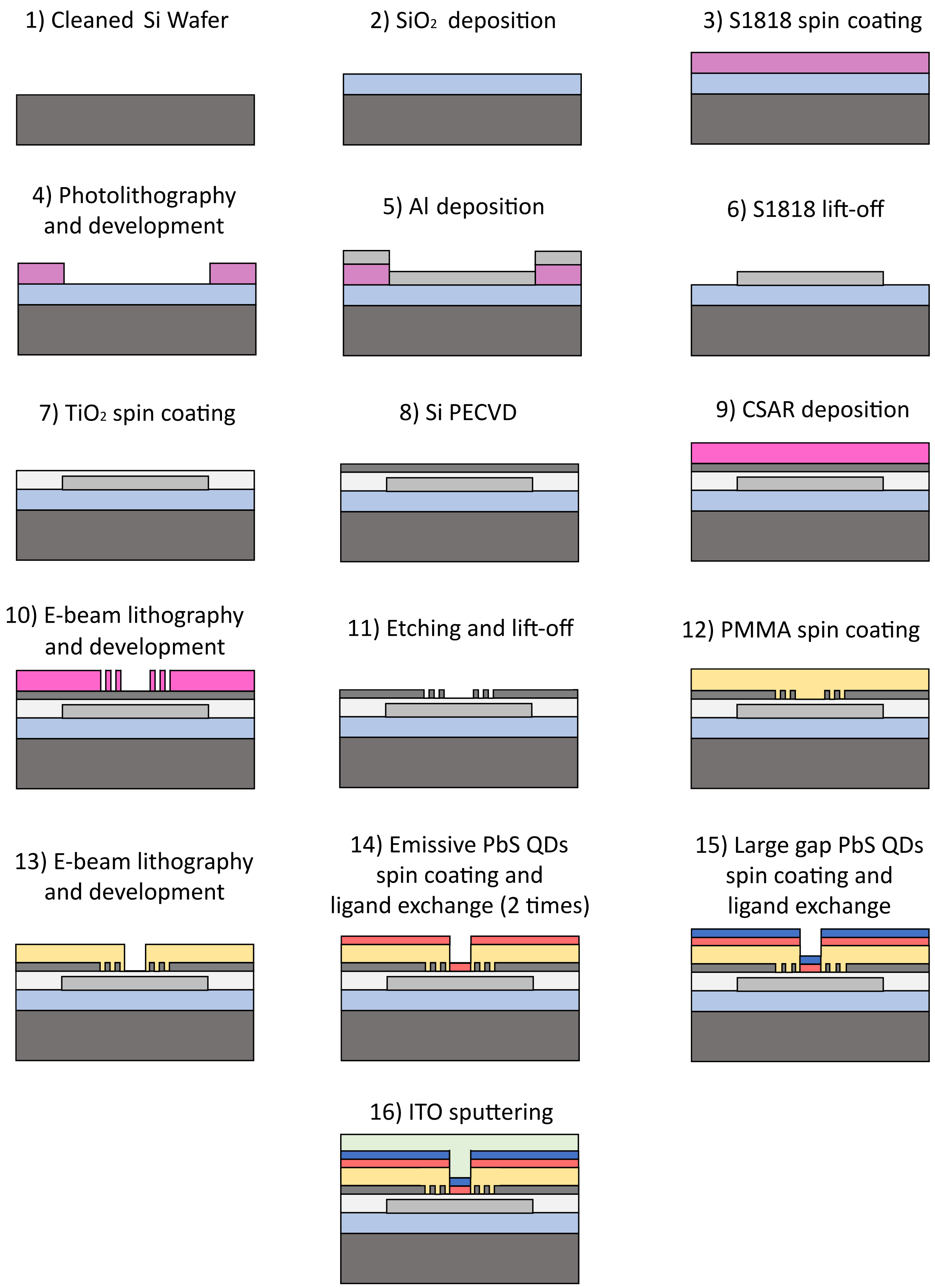

Supplementary Fig. 3 | Fabrication workflow of the QD LEDs operating with an a-Si hologram. The different steps are numbered from 1 to 16.

4. Supplementary data

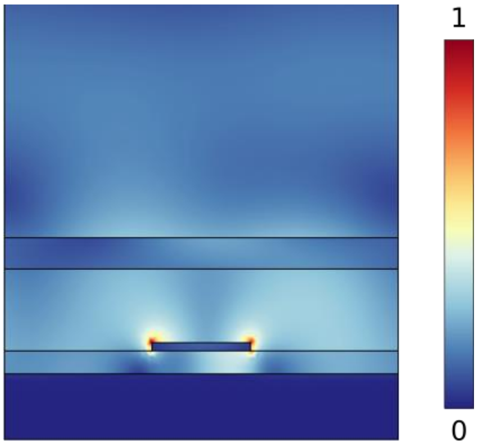

Supplementary Fig. 4 | Full wave simulations showing the norm of the electric field of the TM mode supported by the stack with a Au grating. The field has been evaluated at λ=1520 nm and $k_{//}/k_0$=0.16. The instantaneous H field of this mode is plotted in Fig. 1e.

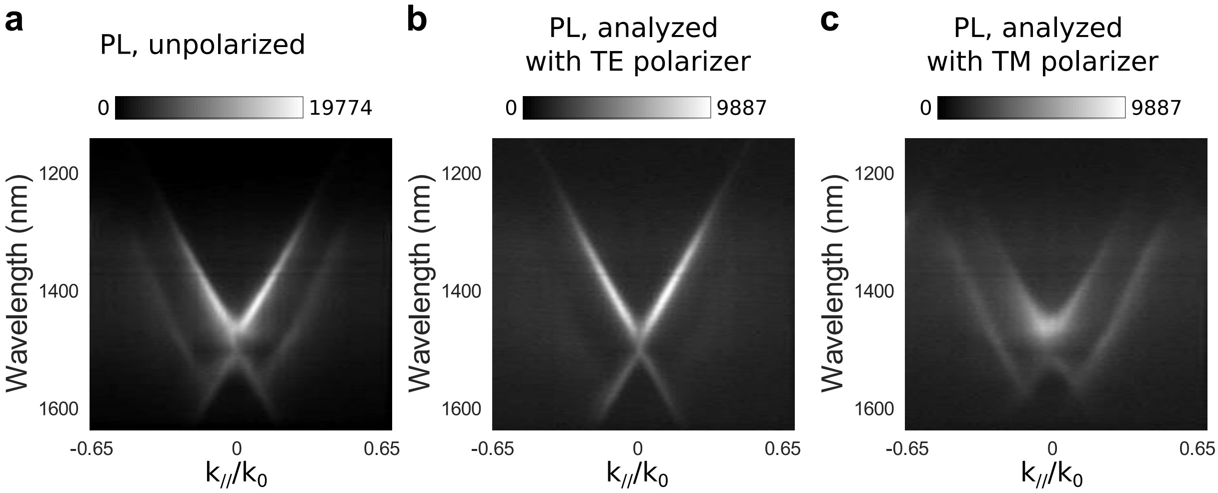

Supplementary Fig. 5 | Photoluminescence experiments on the QD LED with a Au spiral hologram presented in Fig. 1. a Unpolarized dispersion relation. **b** Dispersion relation measured through a polarizer aligned perpendicular to the $k_{//}$ direction. **c** Dispersion relation measured through a polarizer aligned parallel the $k_{//}$ direction.

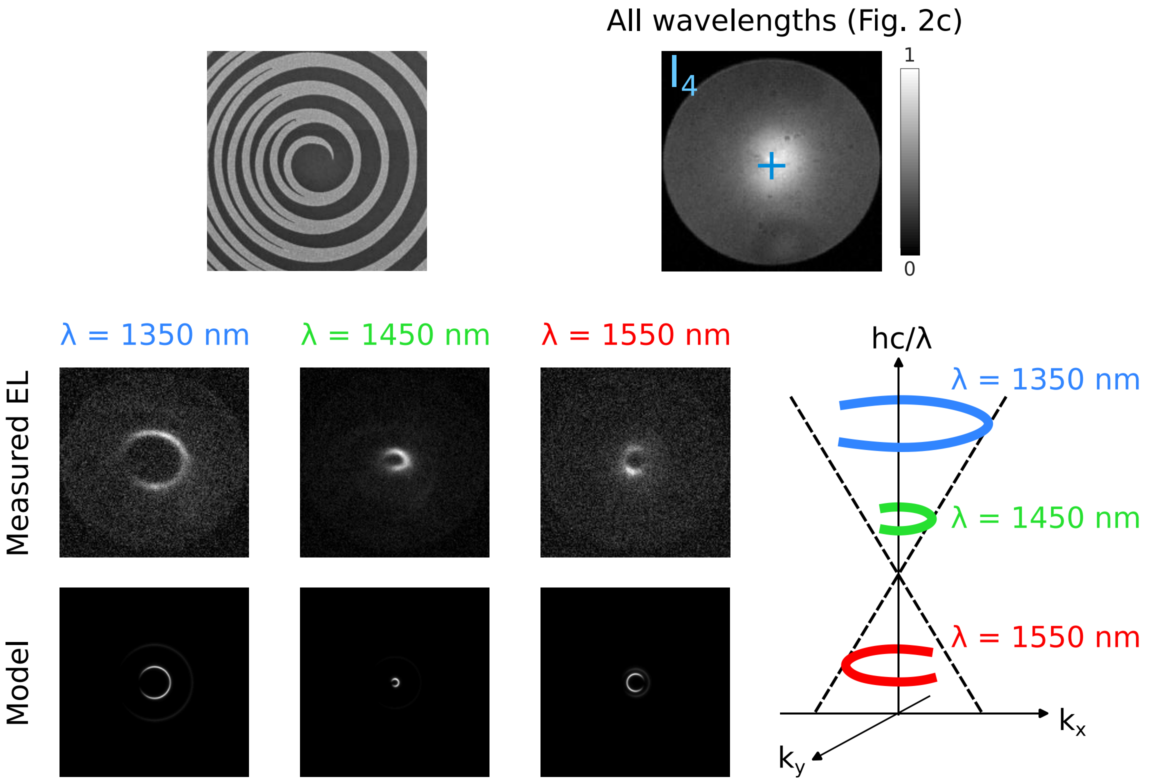

Supplementary Fig. 6 | Extended analysis of the interferometric measurements. We focus here on the interferometric LED producing the I_4 pattern in Fig. 2c. This pattern, which contains all the emitted wavelengths (from $\lambda \approx 1200$ nm to $\lambda \approx 1600$ nm) is reproduced here in the top right panel. To analyze the contribution of individual wavelengths, we have recorded the electroluminescence (EL) of the same device through a succession of three narrow bandpass filters respectively centered on $\lambda = 1350$ nm, $\lambda = 1450$ nm and $\lambda = 1550$ nm (middle row of the figure). The bandwidth is 12 nm for all three filters. Due to the dispersion of the modes supported by the LED (Fig. 1d), the interferometric patterns recorded at different wavelengths are formed at different locations on the sensor. To make sense of these patterns, we remind the reader that the interferences between two monochromatic beams with topological charges *m* and *n* produce an annular pattern with $|m\text{-}n|$ maxima[7]. The crescent-like features evidenced at all three wavelengths is the expected behavior for an annular beam with a topological charge m=1 (i.e. the beam with a phase singularity around which the phase rotates from $-\pi$ to $+\pi$ produced by the spiral) interfering with an annular beam with a topological charge m=0 (i.e. the beam without phase singularity produced by the bullseye): at one point of the annular pattern, there are destructive interferences between the two, while on the other side, there are constructive interferences due to the fact that the phase of the vortex as gained a π factor. These measurements are well reproduced by our simple scalar model (bottom row of the figure) which makes an optical vortex interfere with a directional beam with planar wavefronts. The interference patterns are thinner than the experimental ones because the calculations do not take into account the 12 nm bandwidth of the experimental bandpass filters. Notice that both experiments and calculations show that the contribution of the TE-like mode is much more significant than the TM-like mode (the TM-like mode is essentially hidden in the isotropic background noise of the experimental images, while it is only barely visible as a fainter interferometric crescent in the calculations). Notice also that the orientation of the crescent-like interference pattern changes for $\lambda = 1550$ nm because it is on the other side of the crossing of the TE-like branches (see schematic on the right of the figure).

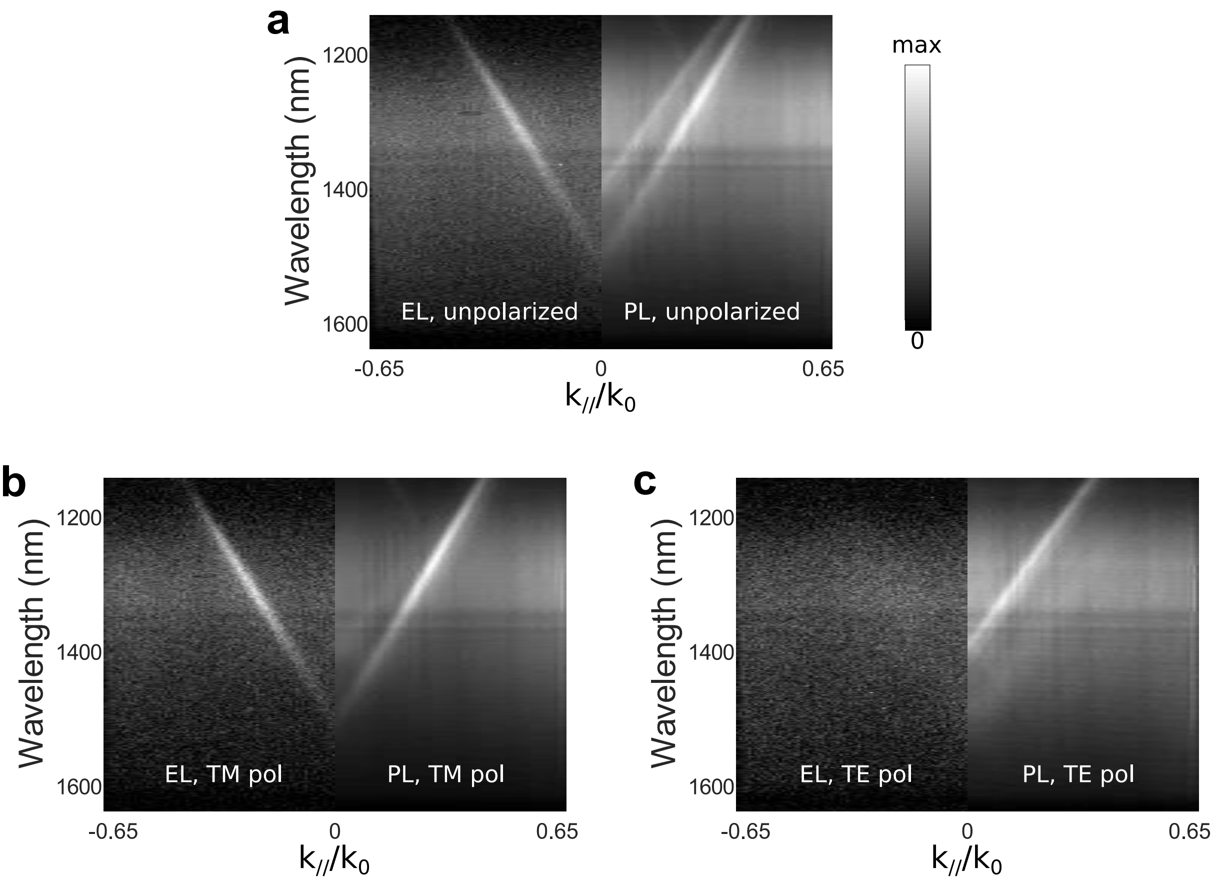

Supplementary Fig. 7 | Polarization analysis of the light emitted by a QD LED operating with an a-Si spiral. a Unpolarized EL and PL dispersion relations (same as Fig. 3e in the main text). **b** EL and PL dispersion relations measured through a polarizer aligned along the $k_{//}$ direction. **c** EL and PL dispersion relations measured through a polarizer aligned perpendicular to the $k_{//}$ direction.

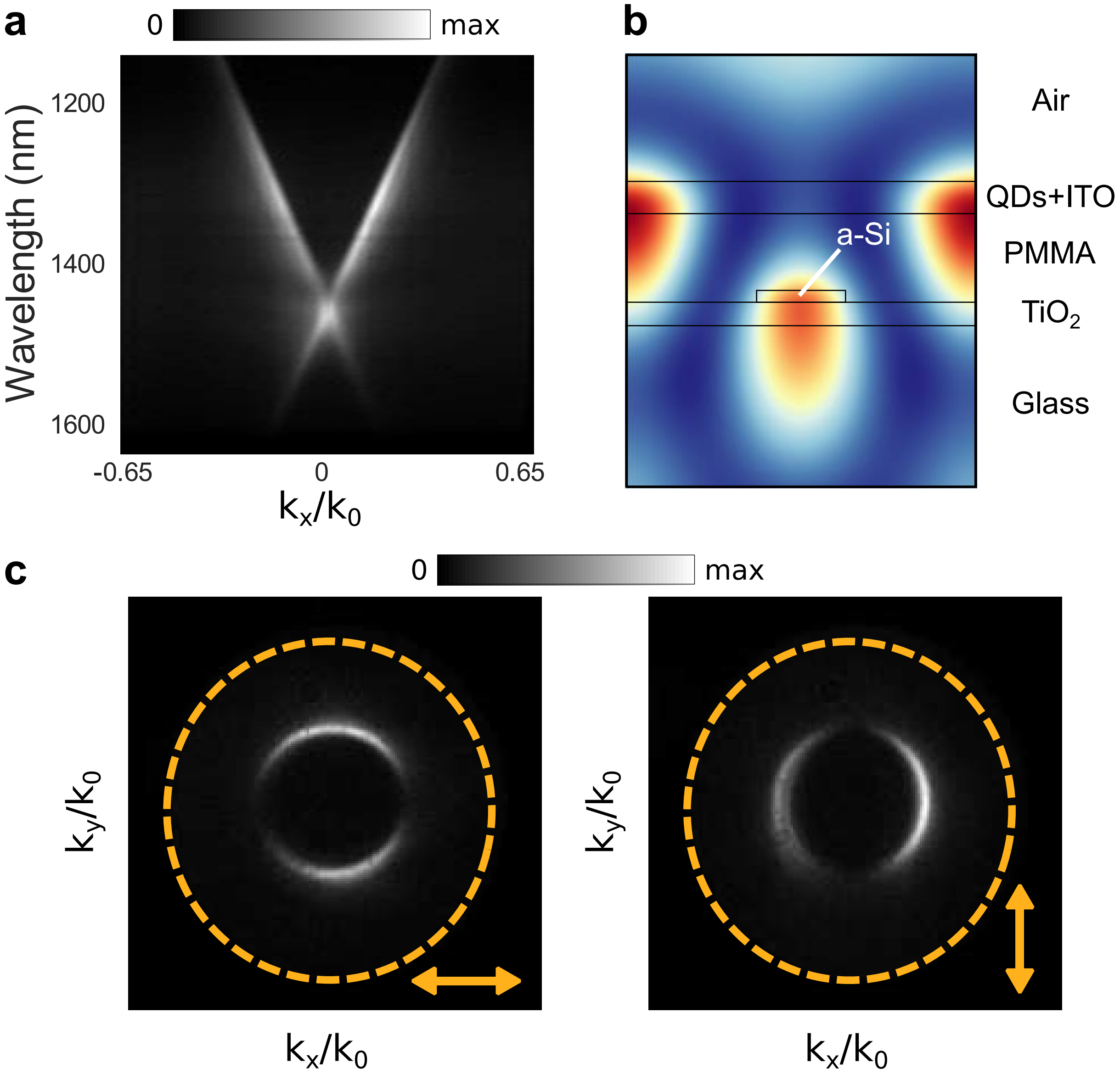

Supplementary Fig. 8 | PL emission properties of the same stack as the QD LED with an a-Si spiral studied in Fig. 3, but without the bottom Al cathode. a Dispersion relation. **b** Finite element simulations of a two-dimensional periodic structure with the same stack as the actual device without bottom Al cathode, representing the square of the electric field norm $|E|^2$. As always in our simulations, we merge the QD layers and the ITO electrode into a single domain with a complex permittivity equal to 4.45+0.13i (see section 1 above). **c** Back focal plane images of the experimental PL at λ = 1250 nm, for two linear polarizations indicated by the double arrows. The yellow dashed circles represent the numerical aperture of the objective, with a radius of 0.65.

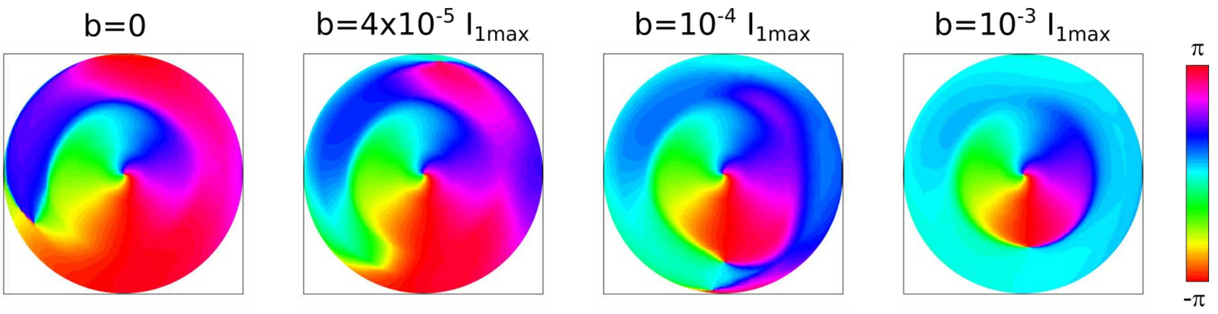

Supplementary Fig. 9 | Assessing the influence of unequal backgrounds in the interferometric images used for the phase evaluation. In our interferometric experiments, the recorded images exhibit an isotropic background corresponding to the light that is not coupled to the modes of interest. This background does not affect the reconstruction of the phase if it reaches the same level in all four interferometric images $I_1,\ldots, I_4$ because it cancels out when evaluating $\arctan[(I_4 - I_2)/(I_1 - I_3)]$. However, Fig. 2c shows that the experimental background of the I_1 image is larger than in the three other interferometric patterns I_2, I_3, I_4 probably due to fabrication imperfections. To assess the influence of this higher background on the accuracy of the phase evaluation, we plot the reconstructed phase predicted by our scalar model when a background constant b is added to each pixel of the computed I_1 image. Four cases are represented, with b expressed as a fraction of the maximum pixel intensity I_{1max} of the computed I_1 image (b=0 means that there is no added background to the computed I_1 image). In all cases, the central singularity is preserved. Only the phase at the periphery of the plot is significantly affected by b. The third case ($b = 10^{-4} I_{1max}$) is the one that gives the best agreement with the experiments and is plotted in Fig. 2e. When integrated over the full numerical aperture, this background per pixel $b = 10^{-4} I_{1max}$ represents 3.6% of the total signal of the computed I_1 image.

5. Supplementary references

1. Lynch, D. W. & Hunter, W. R. Comments on the Optical Constants of Metals and an Introduction to the Data for Several Metals. in *Handbook of Optical Constants of Solids* 275–367 (Elsevier, 1997). doi:10.1016/B978-012544415-6.50015-7.
2. Wang, H. *et al.* Electroluminescence of Colloidal Quantum Dots in Electrical Contact with Metallic Nanoparticles. *Adv. Opt. Mater.* **6**, 1700658 (2018).
3. Rakić, A. D., Djurišić, A. B., Elazar, J. M. & Majewski, M. L. Optical properties of metallic films for vertical-cavity optoelectronic devices. *Appl. Opt.* **37**, 5271 (1998).
4. Wang, H. *et al.* Revisiting the Role of Metallic Antennas to Control Light Emission by Lead Salt Nanocrystal Assemblies. *Phys. Rev. Appl.* **10**, 034042 (2018).
5. Hines, M. A. & Scholes, G. D. Colloidal PbS Nanocrystals with Size-Tunable Near-Infrared Emission: Observation of Post-Synthesis Self-Narrowing of the Particle Size Distribution. *Adv. Mater.* **15**, 1844–1849 (2003).
6. Le-Van, Q., Le Roux, X., Aassime, A. & Degiron, A. Electrically driven optical metamaterials. *Nat. Commun.* **7**, 12017 (2016).
7. Harris, M., Hill, C. A. & Vaughan, J. M. Optical helices and spiral interference fringes. *Opt. Commun.* **106**, 161–166 (1994).
